# Editorial: Beyond ultraviolet B radiation: exploring the impact of UVA on skin, reappraisal of UVA phototherapy, and advances in UVA-damage prevention

**DOI:** 10.3389/fmed.2023.1354131

**Published:** 2024-01-05

**Authors:** Piergiacomo Calzavara-Pinton, Francesco Tonon

**Affiliations:** Dermatology Department, University and ASST Spedali Civili, Brescia, Italy

**Keywords:** UVB, UVA, visible light, phototherapy, photocarcinogenesis

Human skin is hit by a continuous flow of non-ionizing electromagnetic radiation. This flow determines positive effects on human health such as the production of vitamin D, the normalization of keratinization, the isomerization of urocanic acid, the modulation of the acquired immune system, the stimulation of natural immune system responses, acquired pigmentation in melano-competent subjects and effects on the neurological system with increased memory and efficiency of neuromuscular activity (1–4).

Furthermore, exposure to ultraviolet rays both from sun and artificial sources is the oldest and still the most widespread therapeutic tool for many inflammatory skin diseases.

However, on the other hand, exposure to solar radiation is the main cause of photoaging, triggering of photodermatosis and the appearance of both melanoma and non-melanoma skin cancers such as basal cell and squamous cell carcinomas (1, 2).

What is therefore clear is that reducing sun exposure could reduce the appearance of neoplastic pathologies but could also cause serious problems for human health, as well as seriously damaging the quality of life and capacity for social and working life of subjects.

The problem is therefore how we can maximize the beneficial effects of sun exposure and at the same time reduce the risk of adverse ones.

A possible solution lies in better knowledge of the action spectrum and effective dose of skin photobiological reactions and of the opposite photobiological effects of a few wavebands.

An example is that of the production of Vitamin D whose action spectrum is in the UVB waveband but the effective dose for optimal production is much lower than that for erythema and that for squamous cell carcinoma.

In more recent years, attention has shifted first to UVA, both UVA II and UVA I, and then also to the visible, with particular attention to the blue waveband and then also to infrared, which represents the fraction of non-ionizing electromagnetic radiation more abundant in the atmosphere.

In the first work (“*Damaging effects of UVA, blue light, and infrared radiation: in vitro assessment on a reconstructed full-thickness human skin*”) contained in this Research Topic “*Beyond ultraviolet B radiation: exploring the impact of UVA on skin, reappraisal of UVA phototherapy, and advances in UVA-damage prevention*”, Montero et al. report the differential responses in cytokine release with UVA, blue light and infrared radiation in a reconstructed full-thickness human skin. Unlike results of previous investigations, photoaging biomarkers, collagen, metalloproteinases 1 and 9, elastin, and decorin were modulated by UVA and blue light exposure, while not all these markers were affected by infrared radiation. Furthermore, unlike infrared radiation, exposure to UVA and blue light induced loss of fibroblasts and modulation of the photocarcinogenesis markers p53 and p21. In conclusion, the presented results suggest that the various wavelengths of solar light have distinct and differential damaging effects on the skin. These results can give a better improvement of our knowledge of photoaging and photocarcinogenesis and they can be relevant for the formulation of future more effective sunscreens.

Another important issue with very relevant practical consequences has been emphasized by Yang et al. in the article “*The role and safety of UVA and UVB in UV-induced skin erythema*”. They found that comparing the skin irradiated with 2 MEDs of UVB with the skin irradiated with the same dose of UVB combined with UVA, at a ratio UVA:UVB = 8:1, the maximum blood flow depth and blood flow peak of UVB-MED were more increased than UVA+UVB-MED at OCT assessment. Therefore, under the same UVB energy, the skin erythema caused by UVA+UVB was weaker than UVB alone. Therefore, they conclude that the combination of UVA with UVB can have a protective effect on UVB inflammation. These results can be relevant in improving our understanding of the protective effect of UVA against UVB detrimental effects in environmental solar exposure without forgetting, however, that UVA has negative effects itself (5).

Photodermatoses are a well-known adverse effect of sun exposures in many subjects. The most frequent autoimmune (previously called idiopathic) photodermatosis is polymorphic light eruption. It has great inter-individual variability of clinical presentation. In some patients it may occur only in a restricted area of the body surface such as, for example, only on the auricles in children with juvenile spring eruption.

de Gálvez et al. studied clinically, histologically and with phototests five patients with summer and spring elbow rash. Their investigations confirm that the elbow rash is a variant of polymorphous light eruption.

In the last manuscript of this Research Topic, Calzavara-Pinton et al. (“*The realistic positioning of UVA1 phototherapy after 25 years of clinical experience and the availability of new biologics and small molecules: a retrospective clinical study*”) have reviewed the clinical results obtained with repeated medium-dose (50J/cm2) exposures to UVA1 (340–400 nm) radiation in a timeframe of 25 years. The clinical uses changed over time on the basis of the clinical efficacy in comparison to NB-UVB and the emergence of new, safe, and effective drugs, biologics and JAK inhibitors, for conditions such as atopic dermatitis and connective tissue disorders (6). At the same time, they found that UVA1 is often ineffective or minimally effective for some uncommon skin diseases, contrary to previous case reports and small case series. They concluded that although the total number of treated patients declined over time, UVA1 phototherapy continues to be a valuable treatment option for patients with specific skin disorders like morphea, GVHD, granuloma annularis, urticaria pigmentosa and skin sarcoidosis.

## Author contributions

PC-P: Writing – original draft, Writing – review & editing. FT: Writing – original draft, Writing – review & editing.
